# Treatment for Severe Class II Open Bite Using a Bonded Hyrax Expander, IZC Mini-Implants, and MEAW Technique in an Adolescent Patient

**DOI:** 10.1155/2023/8833818

**Published:** 2023-09-25

**Authors:** Mohammad Farahani, Reza Morvaridi Farimani, Fatemeh Eskandarloo

**Affiliations:** ^1^Department of Orthodontics, School of Dentistry, Shahid Beheshti University of Medical Sciences, Tehran, Iran; ^2^Dentofacial Deformities Research Center, Research Institute of Dental Sciences, Faculty of Dentistry, Shahid Beheshti University of Medical Sciences, Tehran, Iran

## Abstract

This case report describes the successful 3D treatment of a patient with a narrow maxilla and a severe class II open bite using a combination of a bonded hyrax expander, infrazygomatic crest mini-implants, and the multiloop edgewise arch-wire (MEAW) technique. A 14-year-old female with a thumb-sucking habit in childhood, presented with a severe open bite, a convex profile, and an obtuse nasolabial angle. Diagnosis revealed a skeletal Class II open bite with moderate crowding in the maxillary and mild crowding in the mandibular arch. Treatment objectives included eliminating the open bite, achieving normal overbite and overjet, and improving upper incisor visibility. Treatment involved the use of a bonded rapid palatal expansion device, mini-implants for maxillary intrusion, fixed appliances, vertical elastics, and a MEAW. Treatment results showed resolution of the open bite, improvement in overbite and overjet, achievement of Class I molar and canine relationships, and improved upper incisors visibility. Fixed appliances were used for the whole 22-month therapy period, and post-treatment records demonstrated that the treatment's objectives were met.

## 1. Introduction

Orthodontic therapy for open bite malocclusion—a space between the upper and lower front teeth when the jaws are closed—has long been considered challenging. Furthermore, the treatment of an open bite malocclusion typically requires a comprehensive approach that addresses not only the teeth but also the jaw and the muscles of the face. Due to these complexities, resolving an open bite malocclusion requires a skilled and experienced orthodontist [1]. Certain orthodontic appliances have been created specifically for the intrusion of the posterior segment. Headgears, multiloop edgewise archwires (MEAW), and vertical elastics are often used for over-erupted maxillary molars. Such procedures can correct open bites, but patient compliance is essential [2]. Thus, when traditional devices are not effective, a surgical approach may be considered as it could be more effective in intruding posterior teeth [3]. However, if the patient is unwilling to undergo surgery, alternative treatment options must be taken into consideration [4].

Rapid maxillary expansion (RME) is a treatment method that has been used to increase the width of the upper jaw [5]. In patients with a skeletal Class I or Class II pattern, RME can cause an increase in the posterior vertical height due to the vertical eruption of the posterior teeth [6]. Since the increase in the posterior vertical dimension is considered a side effect of this treatment the practitioner must evaluate the skeletal pattern, the desired changes, and the potential side effects of RME before starting the treatment [7, 8]. Mini-implants have become increasingly popular for providing secure anchorage for orthodontic tooth movement. To reduce the risk of injury to the root and cessation of movement, it is suggested to use the infrazygomatic crest (IZC) region for skeletal anchorage [9, 10]. The MEAW technique is a method that can be used to effectively close an open bite while also increasing the visibility of the upper incisors [4].

This case report describes the treatment of a patient with a narrow maxilla and severe class II open bite, who was successfully managed using a combination of a bonded hyrax expander, mini-implants placed in the IZC region, and the MEAW technique.

### 1.1. Case Presentation

**Figure 1:**
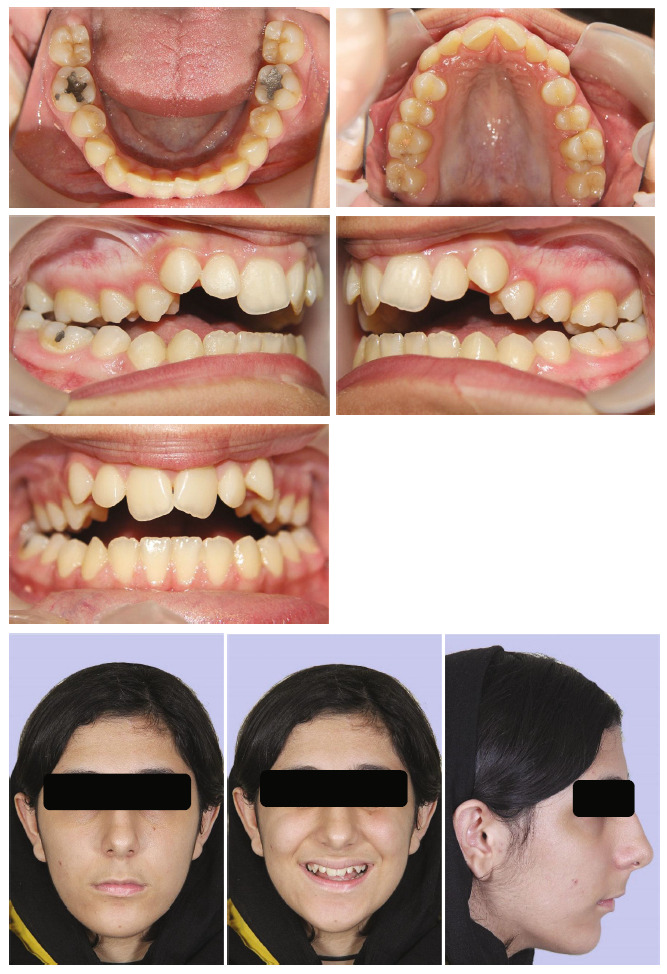
Pretreatment facial and intraoral photographs.

**Figure 2:**
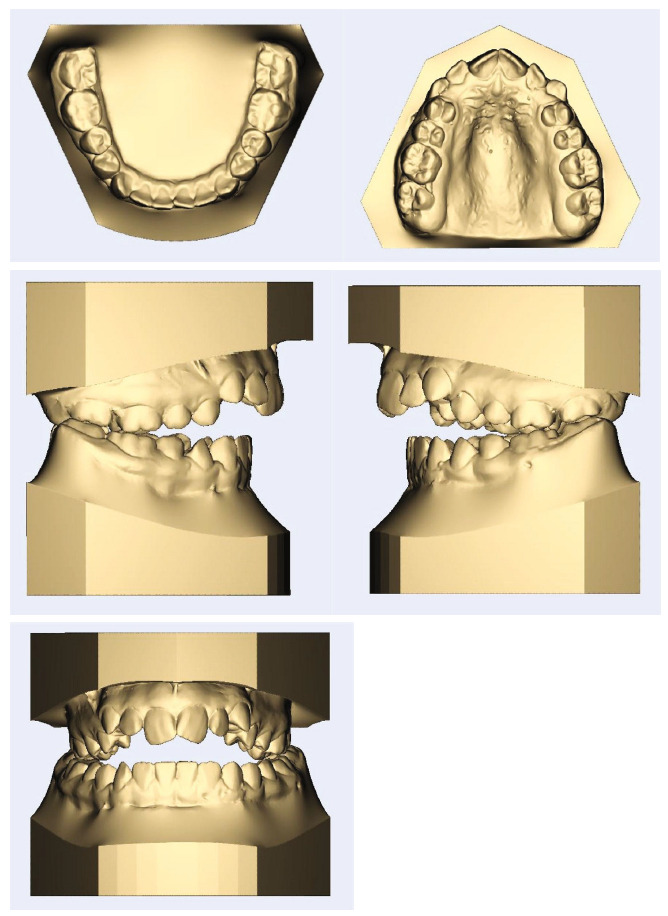
Pretreatment dental models.

The patient, a 14-year-old girl had a significant gap between her upper and lower teeth. In general, she was in good health and had no previous history of major systemic diseases or traumas. Thumb sucking was her childhood habit, and the pattern of tongue trust during swallowing was observed. The patient's soft tissue profile was convex, and the nasolabial angle was obtuse. In the frontal view, a slight chin deviation to the right was observed. Only half of the upper incisors were displayed when smiling. The intraoral examination and dental models indicated a class II molar relationship, approximately 7 mm open bite in the central incisors' region, and 8 mm overjet. Her upper arch had a V shape, and she had a bilateral posterior crossbite with a 7 mm transverse discrepancy in the anterior arch width and 4 mm in the posterior arch width. There was 8 mm crowding in the maxillary arch and 2.5 mm crowding in the mandibular arch. The curve of Spee was increased in both the upper and lower arches. A 3 mm of Bolton discrepancy with the excess of maxillary anterior teeth was measured (Figures 1 and 2). There were no signs or symptoms of temporomandibular joint disorder.

**Figure 3:**
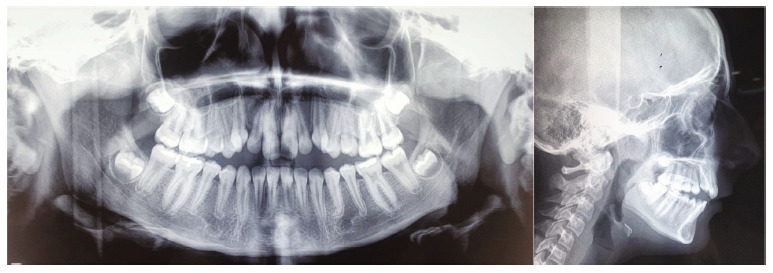
Pretreatment panoramic and cephalometric radiographs.

All third molars were visible in the panoramic radiograph. A Class II skeletal relationship (ANB: 5) and a steep mandibular plane angle (SN-MP: 39) were also obvious, according to the cephalometric analysis. Upper incisors were retro-inclined (U1-SN, 94), and lower incisors were pro-inclined (IMPA, 94; Figure 3; Table 1). With this knowledge in mind, a skeletal Class II open bite was identified as the patient's condition.

The treatment objectives were to eliminate open bite and creation of normal overbite and overjet, obtain Class I molars and canines' relationship, relieve dental crowding, and improve the patient's esthetic by achieving consonant smile arc.

The patient was offered the option of undergoing orthognathic surgery as a treatment after growth cessation. This would consist of advancement and posterior impaction of the maxilla, followed by mandibular autorotation and advancement. She and her parents refused orthognathic surgery at a later time. A second treatment option was to remove the first molars or premolars, followed by retraction of the anterior teeth and posterior protraction during space closure. This treatment was not recommended since it could aggravate the patient's lip retrusion while the anterior teeth retraction. As a third option, intermaxillary elastics were proposed for extruding anterior teeth. It was also excluded due to its relapse tendency and excessive gingival display. As a fourth option, bonded Hyrax for expanding the narrow maxilla, IZC mini-implants for the intrusion of the upper molars, and distalization of the upper arch with a modified MEAW technique to extrude the upper incisors to close the bite and improve the incisor display could be used. Although this alternative was adopted, it was only likely to result in minimal improvements to the profile while addressing the open bite and a slight yet substantial improvement in upper incisor visibility.

**Figure 4:**
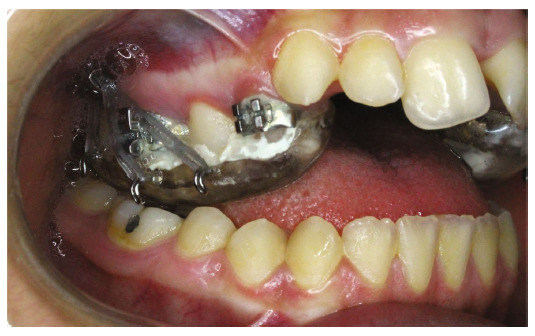
Chain elastics were anchored from bite block hooks to an 8 mm 16 S.S hook-shaped wire put into IZC screws to intrude posterior upper teeth.

Initially, a bonded rapid palatal expansion device with a posterior bite plate containing buccal hooks on the first molar area was cemented in the maxilla. Two 14 mm × 2 mm mini-implants were inserted in the IZC bilaterally. The patient was instructed to turn the screw twice a day. The upper arch expanded by 8 mm during the course of 16 days, and the crossbite in the posterior region was corrected. After the expansion was completed and during stabilization, chain elastics were anchored from the hooks embedded into the bite block to an 8 mm 16 S.S hook-shaped wire that was inserted into IZC screws to intrude the posterior upper teeth (Figure 4). Since the clinic was closed due to the COVID-19 outbreak, the patient was not visited for 4 months, and the chains were not changed. Afterward, new elastomeric chains were applied, and the expander device was removed one month later.

**Figure 5:**
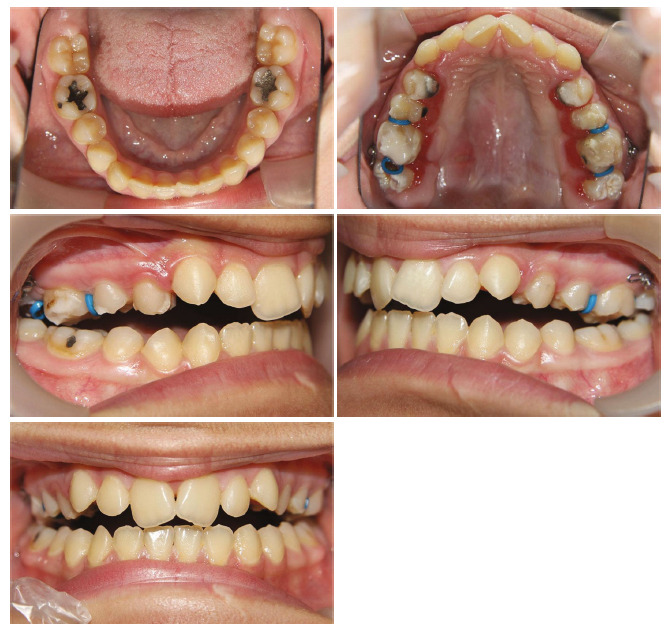
The open bite reduced after removal of the expander.

The open bite was then closed partly in the region of the central incisors, whereas reduced to 5 mm in the region of the canines (Figure 5). The patient was bonded with a straight wire fixed appliance in the upper dental arch (0.018^″^ × 0.025^″^ slot, American orthodontics), and upper second molars were bonded at the next session. The fixed appliance was placed in the mandibular dental arch three months after the upper arch. Leveling and alignment were started with the use of 0.012 Ni–Ti, followed by 0.014 Ni–Ti, 0.016 Ni–Ti, and 0.016 stainless steel wires. Unwanted hanging of the upper second molars palatal cusps has occurred, and we decided to insert two 9 mm × 1.6 mm mini-implants bilaterally in the palate between the first and second maxillary molars to intrude second upper molars. After completion of the intrusion, some amount of anterior open bite remained.

**Figure 6:**
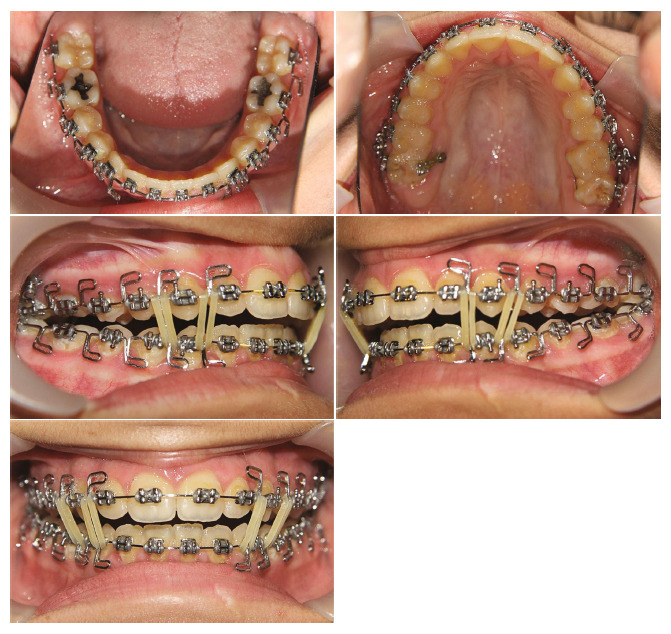
MEAW arches with 0.016^″^ × 0.022^″^ S.S. wires and elastics 1/8″, 6 oz.

**Figure 7:**
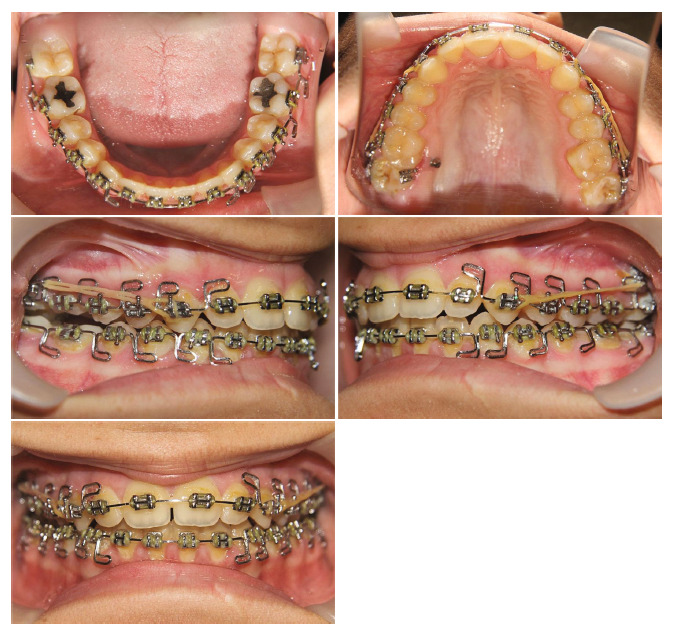
Chain elastics acting from the upper canines to the IZC screws.

A MEAW using 0.016 × 0.022 stainless steel wire was inserted (Figure 6). The anterior open bite was corrected by patient compliance and using vertical elastics (1/8″, 6 oz) in two months. The Class I relationship was obtained using chain elastics acting from the upper canines to the IZC screws on both sides (Figure 7). Final detailing of the occlusion was completed at the finishing step using 0.017 × 0.025 stainless steel wire in the upper arch and 0.016 stainless steel wire with a reverse curve of Spee in the lower arch. The treatment took 22 months with fixed appliances. After the course of treatment was complete, the brackets were taken off, and vacuum-formed and lingual fixed retainers were inserted in the upper and lower arches, respectively.

**Figure 8:**
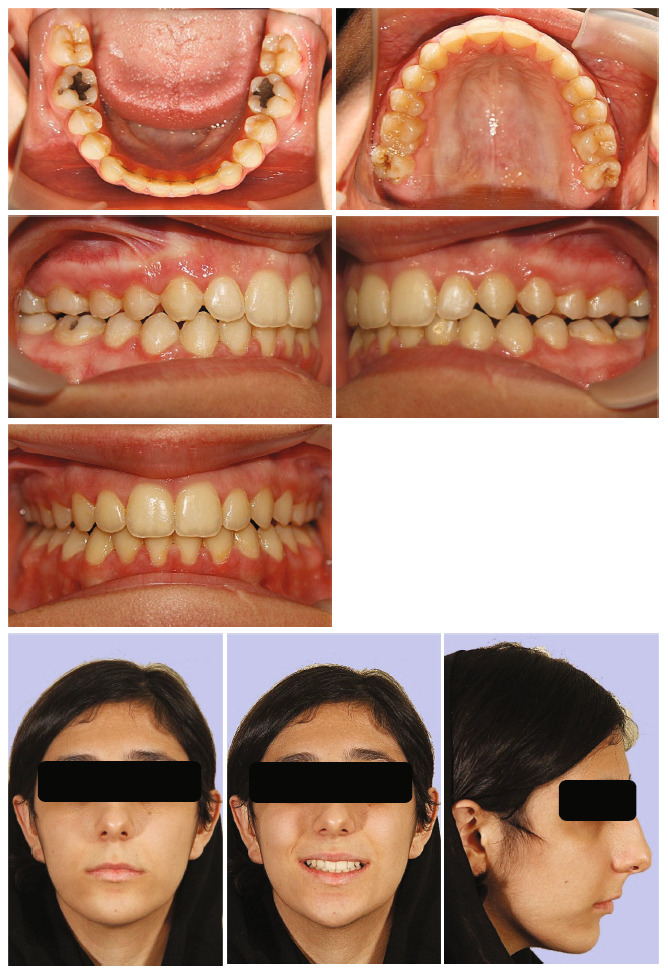
Posttreatment facial and intraoral photographs.

**Figure 9:**
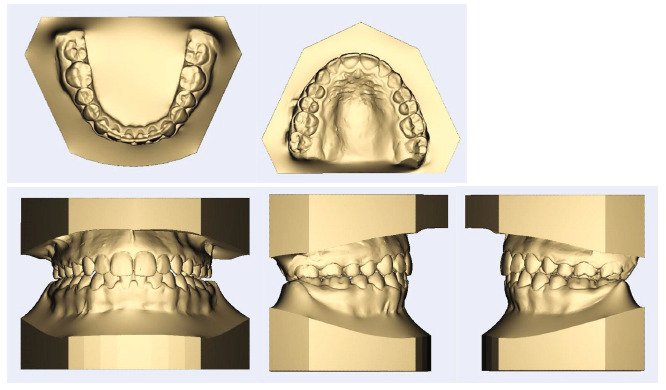
Posttreatment dental models.

**Figure 10:**
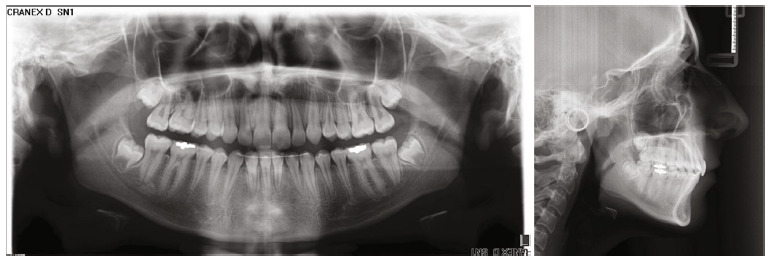
Posttreatment panoramic and cephalometric radiographs.

**Figure 11:**
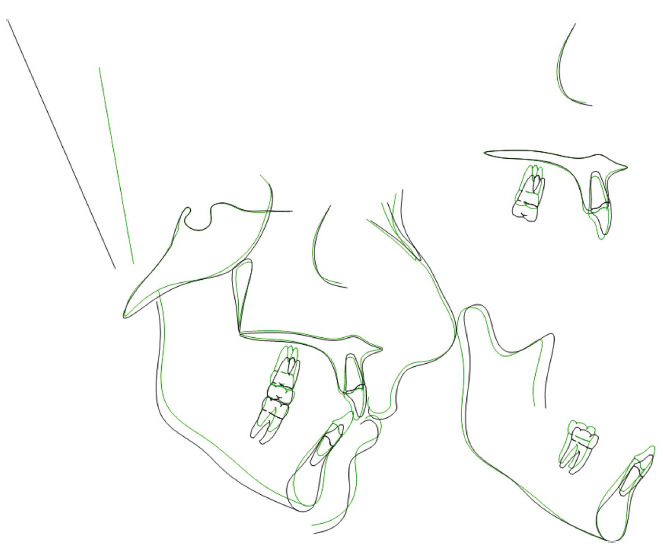
Superimposition at pretreatment (black) and posttreatment (green).

The posttreatment records demonstrated that the treatment goals had been met in all three dimensions (Figures 8 and 9). The smile arc in the facial photographs became consonant. The patient's chief complaint, the open bite, was resolved, and a respectable overbite and overjet were obtained. Intraoral records showed Class I molar and canine relationships and the posterior crossbite was eliminated. The panoramic radiograph showed adequate root parallelism. The posttreatment cephalometric tracing and superimposition revealed a rotation of the mandible and a decrease of 1.7° in the mandibular plane angle. Observations indicated a significant intrusion of the maxillary molars (5.4 mm) and an increase in the visibility of the upper central incisors (from −1 to +3 mm; Figures 10 and 11; Table 1).

## 2. Discussion

The treatment of open bite malocclusion can be complex and challenging due to the multifaceted nature of its underlying causes and the need for a comprehensive approach that addresses not only the teeth but also the jaw and the muscles of the face. Traditional treatment methods, such as headgear and MEAW with vertical elastics, may not always be successful, and a surgical approach may be considered if the patient is willing to undergo surgery. However, in cases where surgery is not desired, alternative treatment options need to be considered [1, 11].

At first glance, our patient was presented with an extreme Class II open bite and maxillary constriction, requiring surgical intervention. Nonetheless, the open bite contributed in part to a thumb-sucking propensity that resulted in protrusion and intrusion of the incisors. Considering this, a well-designed and meticulously implemented orthodontic method could be a viable alternative to surgical correction. To correct the open bite, noninvasive procedures, such as molar intrusion or incisor extrusion, could be considered [2]. In addition, to resolve the Class II relationship of the teeth, extraction or distalization may be performed; however, since the patient's lips were already retruded, extraction may have adverse effects on the soft tissue profile [12]. RPE was chosen for maxillary constriction because the patient was still of age to undergo sutural expansion.

Mini-implants and mini-plates can be used as absolute intraoral anchorage units for molar intrusion to mitigate the need for patient compliance [13]. Mini-implants have additional benefits including low cost, simple insertion and removal techniques, the ability to be inserted in multiple areas of the alveolar process and basal bone, simplicity of cleaning, enhanced orthodontic mechanics, and high patient acceptance. Nonetheless, proper orthodontic planning and management, the selection of the optimal insertion site based on specific criteria, and the execution of an ideal surgical approach are required [14].

To obtain the maximum intrusive vector on the posterior teeth, we designed a simple Hyrax with three hooks on each side of the appliance. In addition, using a highly rigid structure, such as Hyrax, allowed us to use the minimum number of mini-implants (one on each side's buccal) with the highest efficacy for symmetrical intrusion. Mini-implants are intended to be inserted in the IZC region because, unlike interradicular mini-implants, IZCs do not pose a threat to the roots and are more stable [9, 15]. After bonding the patient's fixed appliances, the maxillary plan alteration continued with the addition of a distal vector. From a biomechanical standpoint, a line of distal force is applied from the IZCs to the maxillary arch using chain elastics on the canines pass below the center of resistance point. This aligns with our goals, as it could lead the maxillary plane to steepen and clinically result in bite closure and increased visibility of upper incisors. The patient's overbite changed from −7 to +2 mm at the end of treatment, confirming our strategy was effective. Consequently, simultaneous total arch distalization and posterior intrusion of the maxillary dentition were accomplished, enhancing both the smile aesthetics and occlusion.

It has been demonstrated that MEAW therapy is effective in treating open bite cases. Long-term follow-ups in earlier studies assessing the stability of this technique failed to find any significant recurrence [16]. There is disagreement in the literature on the MEAW's efficacy for the intrusion of posterior teeth, even though it is helpful for uprighting the buccal segments and retraction or extruding front teeth [4, 17, 18]. MEAW therapy is further constrained by how heavily it requires compliance from patients to be effective. The root resorption that occurs in individuals whose incisors have been vertically moved raises yet another issue with employing this method [19]. No root resorption was observed on either the molars or the incisors as a result of combining posterior intrusion with TADs and MEAW to minimize the vertical movement of the teeth. The extrusion of the upper incisors is important not only for correcting the open bite but also for improving smile esthetics with the fewest possible side effects, which was greatly aided by the patient's use of the MEAW mechanic (Figure 10).

This orthodontic treatment not only fixed the open bite but also altered the profile, as shown by the slight advancement in the chin (Figures 3, 10, and 11). The intrusion of the upper molars, a characteristic also noted in earlier investigations, may have caused a favorable mandibular rotation that led to this. However, it has been claimed that the chin advancement that results from mandibular counterclockwise rotation is case-sensitive and limited [20–22]. To further advance the chin and lessen the nasal hump for this patient, genioplasty and rhinoplasty surgeries were recommended after the orthodontic treatment.

It was typically perceived appropriate to close the bite and restore buccal occlusion exclusively using elastics. On the left side, however, there were modest openings demonstrating an imperfect occlusion (Figures 8 and 9). These gaps were challenging to correct in part because of the slight rightward deviation of the mandible that was apparent at the commencement of treatment. In addition, there was insufficient bone space to distalize the left buccal segment because the patient declined surgery to remove the upper third impacted molars. The absence of rebound and worsening of the bite in the buccal segments one month after the patient ceased using box elastics on the left and right side was promising.

In managing patients with complex and severe malocclusions, it is crucial for orthodontists to carefully consider all available treatment methods, weighing their pros and cons. Orthodontists can broaden treatment options while reducing morbidity and expense by combining approaches. However, sometimes complex orthodontic treatments can be done non-surgically at the cost of time and more expenses. In this case, synchronizing expansion with posterior intrusion and using biomechanics like clockwise maxillary plane rotation were beneficial. Root resorption was prevented, and treatment time was shortened, which is crucial for adolescent patients. However, the patient's non-compliance prevented post-treatment records from being kept. Despite this limitation, this case report indicates that the utilization of a multidisciplinary approach and evidence-based techniques can empower orthodontists to effectively manage complex cases and achieve favorable treatment outcomes for their patients.

When used in combination with a Hyrax expander, IZC mini-implants were found to be helpful for the intrusion of posterior teeth, shorten treatment duration, and prevent the expander's negative effects in the vertical dimension. The open-bite closure was improved and completed with the use of the MEAW approach, which also raised the visibility of the upper incisors and improved the aesthetics of the patient's smile.

## Figures and Tables

**Table 1:** Cephalometric analysis.

Measurement	Norm	Initial	Final
SNA (°)	81.0	77.0	77.0
SNB (°)	79.0	71.0	72.7
ANB (°)	2.0	5.1	4.7
Inclination angle (°)	85.0	86.5	86.3
Wits appraisal (mm)	−1.0	5.5	−1.2
SN to MP (°)	34.0	39.7	38.0
U1 to SN (°)	102.0	93.8	90.4
L1 to MP (°)	90.0	93.7	92.6
Upper lip to E-line (mm)	−1.0	−3.5	−3.0
Lower lip to E-line (mm)	0.0	−3.1	−4.0

## Data Availability

The diagnostic records of the patient are available.
